# PANTHER version 16: a revised family classification, tree-based classification tool, enhancer regions and extensive API

**DOI:** 10.1093/nar/gkaa1106

**Published:** 2020-12-08

**Authors:** Huaiyu Mi, Dustin Ebert, Anushya Muruganujan, Caitlin Mills, Laurent-Philippe Albou, Tremayne Mushayamaha, Paul D Thomas

**Affiliations:** Division of Bioinformatics, Department of Preventive Medicine, Keck School of Medicine, University of Southern California, Los Angeles, CA 90033, USA; Division of Bioinformatics, Department of Preventive Medicine, Keck School of Medicine, University of Southern California, Los Angeles, CA 90033, USA; Division of Bioinformatics, Department of Preventive Medicine, Keck School of Medicine, University of Southern California, Los Angeles, CA 90033, USA; Division of Bioinformatics, Department of Preventive Medicine, Keck School of Medicine, University of Southern California, Los Angeles, CA 90033, USA; Division of Bioinformatics, Department of Preventive Medicine, Keck School of Medicine, University of Southern California, Los Angeles, CA 90033, USA; Division of Bioinformatics, Department of Preventive Medicine, Keck School of Medicine, University of Southern California, Los Angeles, CA 90033, USA; Division of Bioinformatics, Department of Preventive Medicine, Keck School of Medicine, University of Southern California, Los Angeles, CA 90033, USA

## Abstract

PANTHER (Protein Analysis Through Evolutionary Relationships, http://www.pantherdb.org) is a resource for the evolutionary and functional classification of protein-coding genes from all domains of life. The evolutionary classification is based on a library of over 15,000 phylogenetic trees, and the functional classifications include Gene Ontology terms and pathways. Here, we analyze the current coverage of genes from genomes in different taxonomic groups, so that users can better understand what to expect when analyzing a gene list using PANTHER tools. We also describe extensive improvements to PANTHER made in the past two years. The PANTHER Protein Class ontology has been completely refactored, and 6101 PANTHER families have been manually assigned to a Protein Class, providing a high level classification of protein families and their genes. Users can access the TreeGrafter tool to add their own protein sequences to the reference phylogenetic trees in PANTHER, to infer evolutionary context as well as fine-grained annotations. We have added human enhancer-gene links that associate non-coding regions with the annotated human genes in PANTHER. We have also expanded the available services for programmatic access to PANTHER tools and data via application programming interfaces (APIs). Other improvements include additional plant genomes and an updated PANTHER GO-slim.

## INTRODUCTION

**Figure 1.**
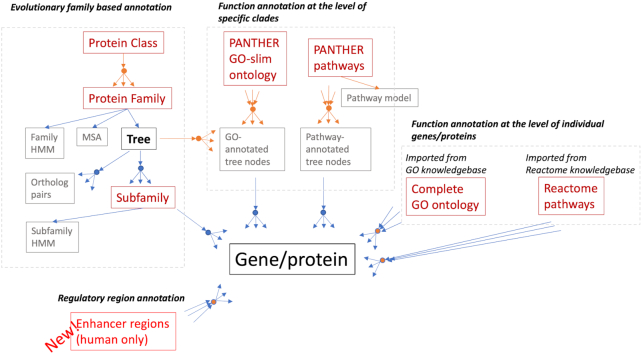
Relationships between classifications and data in PANTHER, showing the different ways in which a gene/protein (bottom) can be classified (red), as well as the other data available in PANTHER (gray). Classification types are shown in red. Arrows indicate the nature of the relationship (1:1, 1:many, many:many) and how the relationships are derived (blue = computationally, orange = expert curation, orange circle in blue = both methods). As a result, a given gene can be associated with only one subfamily, family and protein class, but can be associated with more than one GO term, pathway or enhancer region.

PANTHER (Protein Analysis Through Evolutionary Relationships) is an integrated knowledgebase of evolutionary and functional relationships between protein-coding genes (1) (referred to hereafter as proteins), as well as tools for using the classifications to analyze large-scale genomics data (2,3). Proteins are classified along two primary axes (Figure 1): evolutionary groupings (Protein Class, Protein Family, Subfamily), and functional groupings (Gene Ontology and pathways). Evolutionary groupings correspond to the ‘natural classification’ of proteins according to their evolutionary histories. Protein families are groups of proteins that are homologous (share a common ancestor) and can be aligned to one another in a multiple sequence alignment that passes some basic quality checks. Subfamilies are orthologous groups of proteins identified in the phylogenetic tree inferred for a given protein family. There can be many subfamilies per family, and some families are very diverse. The subfamilies identified by PANTHER are an important feature that give the annotations a high degree of specificity. At a more general level, protein families are grouped together into broad functional classes (Protein Class).

Functional groupings classify proteins according to the functions of individual proteins, not families. Functions are classified in multiple ways: three different aspects defined by the Gene Ontology (4,5) (molecular function, cellular component, and biological process), and pathways (signaling and metabolic pathways). Functions are annotated using two different methods. The first method annotates clades (defined using internal tree nodes) of related proteins in a phylogenetic tree, resulting in annotations of all the proteins in the annotated clade. These annotations include Gene Ontology terms from PANTHER GO-slim, a simplified or ‘slim’ subset of the complete GO, as well as terms from the PANTHER Pathway (6) ontology. The PANTHER Pathway module (6) includes assignments of protein clades to pathway components, as well as a pathway model generated using CellDesigner (7), which can be displayed or downloaded. The second method annotates individual proteins to functional classes, either the ‘complete’ Gene Ontology (imported from the GO resource), or the Reactome pathways (8) (imported from the Reactome resource).

One of the primary features in PANTHER is a set of reference phylogenetic trees. PANTHER provides extensive data for each protein family:

- A phylogenetic tree
- GO annotations for internal nodes of the phylogenetic tree (available at pantree.org)
- Hidden Markov models (HMMs) for each family and subfamily
- Multiple sequence alignment (MSA) for all family members
- Pairwise orthologs, within-species paralogs, and xenologs

Reference trees are currently available for over 15 000 protein families, and include all members of each family in 142 fully sequenced genomes. These genomes sample the entire tree of life, though the selection of genomes is biased toward ‘model’ organisms for which extensive experimentally-supported GO annotations have been captured by the GO Consortium: four vertebrates (human, mouse, rat, zebrafish), two invertebrates (fruit fly and the nematode worm *C. elegans*), two single-celled fungi (‘yeasts’: budding yeast and fission yeast), the slime mold *D. discoideum*, the plant *A. thaliana*, and the bacterium *E. coli*. Sampling of plant genomes has also recently been expanded. Table 1 shows the sample of organisms in PANTHER version 16 reference trees.

**Table 1.** Number of genomes from each kingdom/phylum in PANTHER 16.0 phylogenetic trees

Taxonomic group	Number of genomes
Bacteria	35
Archaea	8
Fungi	14
Plants	40
Protista and alveolata	8
Amoebozoa	3
Invertebrates	15
Vertebrates	19
Total	142

As described in more detail below, we have also recently added the PEREGRINE database of gene-enhancer links [submitted]. Users can now view lists of enhancers that have been associated with the expression of each human protein-coding gene in PANTHER. Users can also upload a list of genomic regions to PANTHER and retrieve a list of genes linked to enhancers in those regions.

Finally, PANTHER provides three main types of software tools: (i) protein classification tools, (ii) gene list analysis tools and (iii) a protein coding variant analysis tool. The protein classification tools enable users to apply the PANTHER classifications to their own protein sequences of interest. PANTHER now has two classification tools: PANTHER HMMs, and TreeGrafter. PANTHER HMMs use family and subfamily-level HMMs to classify user-specified protein sequences (2,9), and have also been integrated into InterProScan (10). TreeGrafter is a new tool (described in more detail below) that utilizes the PANTHER trees for classification (11), localizing a user-specified sequence to its most likely branch in the tree. Because the PANTHER trees have been annotated with both subfamily names (subtrees) as well as GO terms describing functions, TreeGrafter can also assign subfamilies and GO functions. The gene list analysis tools enable users to upload lists of genes or proteins and perform statistical tests to find enriched functional classes in their list (2,3). The over-representation tool takes an input list of genes and reports statistically over- and under-represented classes relative to a reference list. The enrichment tool takes an input list of genes and quantitative values (e.g. expression levels) and reports classes for which the gene values are distributed non-randomly (e.g. tend to be higher or lower than average). In addition to the 142 genomes in the reference trees, PANTHER has pre-computed annotations for hundreds of other genomes, which are also available for analysis on the PANTHER website.

For over 20 years, PANTHER has supported a large user community in various capacities. PANTHER maintains collaborations with core data resources and consortia. As a member of the Gene Ontology Consortium, PANTHER family trees are the backbone of the GO phylogenetic annotation effort (12,13,14). In addition, PANTHER gene list analysis tools serve as the backend tool for the GO enrichment tool. PANTHER also collaborates with UniProt (15), InterPro (16), and Phylogenes (http://www.phylogenes.org/). PANTHER tools are highly used by the scientific community, many of whom are bench scientists. According to Google Analytics, 170 838 unique users visited the PANTHER website this year (1/1/2020–9/8/2020), compared to 144 572 for the same period in 2018.

Here, we give an overview of the taxonomic coverage of PANTHER annotations, and report the improvements made to PANTHER since our last update two years ago (13). Improvements have been made in several areas: the protein families, the classifications (particularly Protein Class, Protein Family, and PANTHER GO-slim), new web services via application programming interfaces (APIs), new software tools, and the addition of enhancer data.

### Coverage of PANTHER annotations for different taxonomic groups

**Figure 2.**
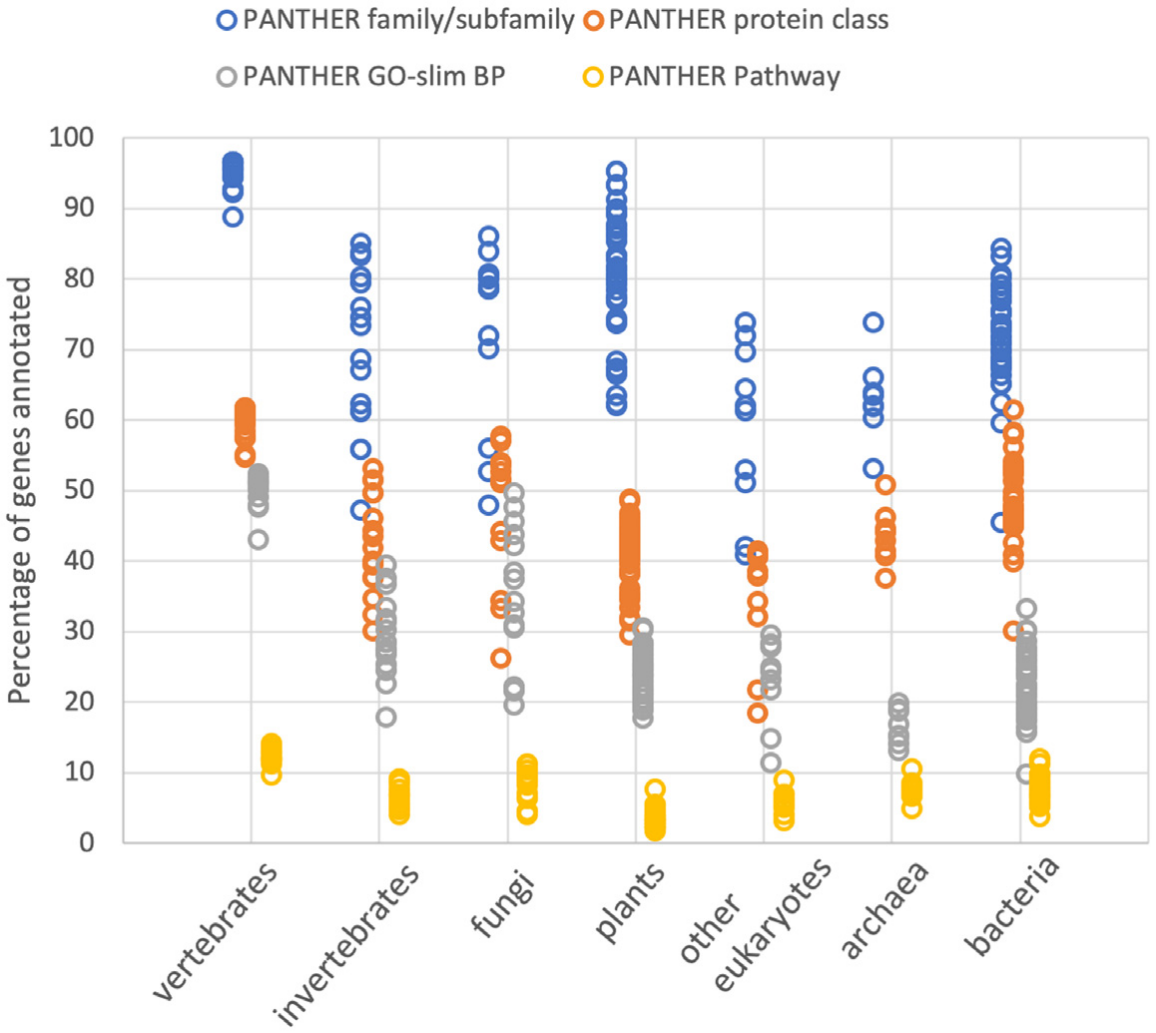
Coverage of protein-coding genes by PANTHER evolutionary and clade-level annotations. Each circle represents the coverage of one of the 142 PANTHER reference genomes, for different annotation types. Vertebrate genomes have the highest coverage for all annotation types, but other genomes are still covered to an appreciable extent.

PANTHER has continued to expand coverage of the 142 genomes represented in the phylogenetic trees, and many clade-specific families have been added to PANTHER to increase coverage of these genomes. Nevertheless, as discussed above, the sampling is biased toward genomes that are relatively well-characterized experimentally. As a result of this uneven sampling, the current coverage of genes by PANTHER annotations varies somewhat for different genomes (Figure 2). PANTHER family and subfamily annotations are highest for vertebrates (over 90% on average), slightly lower for plants (over 80% on average), and slightly lower still for invertebrates, fungi and bacteria (over 70% on average). Not surprisingly the lowest coverage is for single-celled eukaryotic species and for archaea, although coverage is still fairly high (usually 50–70%). Other family- and clade-level annotation types have lower coverage than families and subfamilies (Figure 2). Thus, PANTHER analysis tools can be applied broadly to genomes of organisms across the tree of life, though annotations are generally more complete for those that are more closely related to a well-studied ‘model’ organism (*viz*. vertebrates, insects, nematodes, ascomycete fungi, dicot plants and gamma-proteobacteria; bacilli are also relatively well sampled among bacteria).

**Figure 3.**
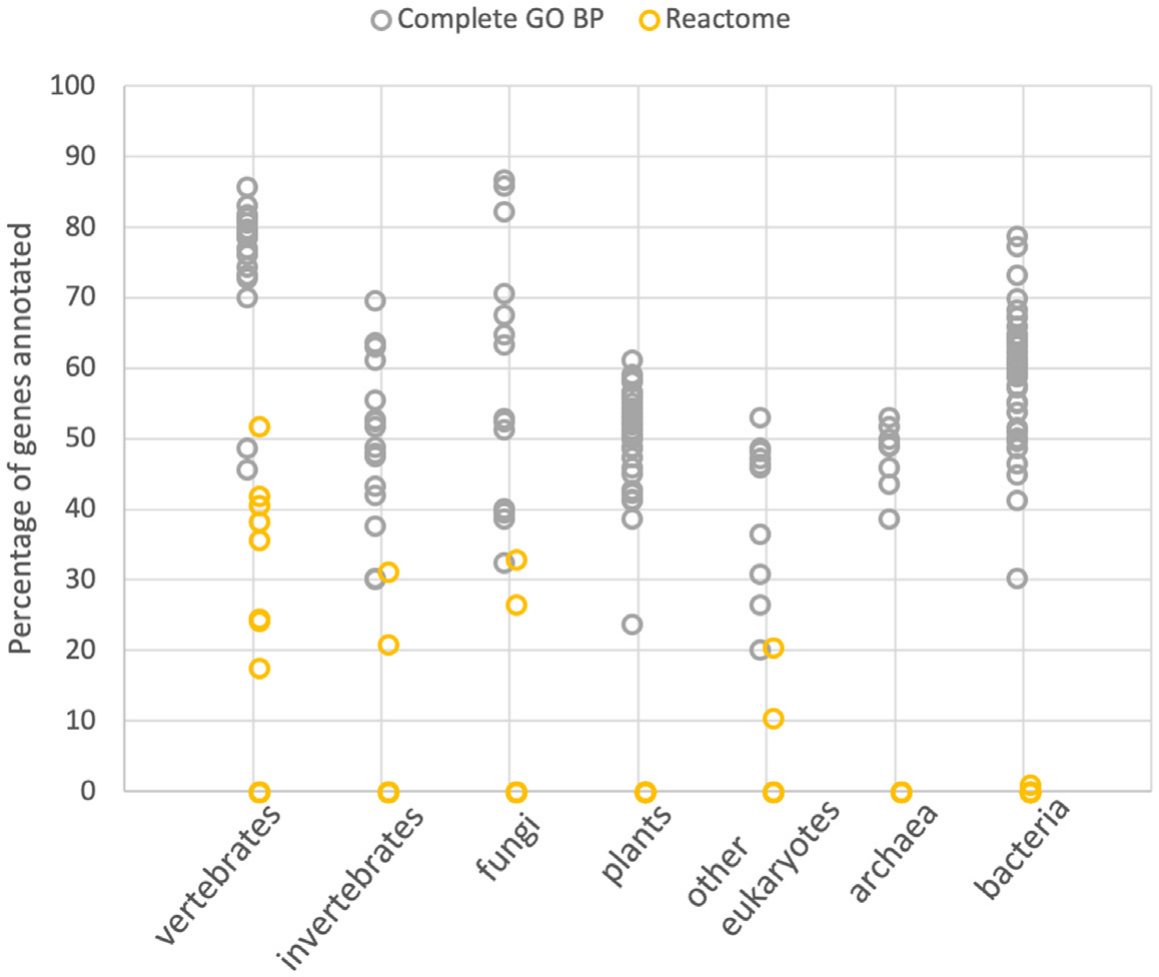
Coverage of protein-coding genes by individual protein-level annotations imported from GO and Reactome. Each circle represents the coverage of one of the 142 PANTHER reference genomes, for different annotation types. Coverage is more variable than for evolutionary and clade-level annotations, and most genomes are not covered by Reactome annotations (overlapping points at 0).

The coverage of individual gene-level annotations depends even more strongly on the organism being analyzed (Figure 3). GO ‘complete’ annotations, imported from the GO database, are assigned by many different methods including manual annotation from the literature, manually reviewed homology inferences (including the PANTHER GO-slim annotations) and computational methods such as InterPro2GO (17). Reactome pathway annotations are imported as well, and cover genes in humans and a few other selected organisms based on orthology relationships to human genes.

### Improved protein families

PANTHER families are constructed each year, using the latest protein sequences available from UniProt Reference Proteomes (15,18). These proteomes are ‘gene-centric,’ meaning that there is a single representative protein sequence for each protein-coding gene in each of the selected genomes. Consequently, the protein families can also be interpreted as protein-coding gene families. Proteins (tree leaves, or extant genes) and internal tree nodes (ancestral genes) are given stable identifiers that are forward-tracked between releases. Since our last update, we have added 10 more plant genomes to PANTHER, in order to improve the phylogenetic representation of plant gene phylogeny. The complete list of genomes in PANTHER trees can be found at http://pantherdb.org/panther/summaryStats.jsp.

The addition of these plant genomes resulted in a significant increase in the number of genes for some families that have been highly duplicated in plants. Some of these families became much more diverse, affecting the alignment quality and therefore the phylogenetic trees, as well as causing difficulties in visualizing and navigating them. We therefore performed manual curation to split these trees into smaller and more well-defined families. As a result, there is a slight increase in the number of families (Table 2).

**Table 2.** Comparison of numbers of genomes, genes and families between PANTHER 14 and 16

	PANTHER 14.1 (2018)	PANTHER16.0 (2020)
Genomes	132	142
Genes in PANTHER families (trees)	1 750 742	2 065 831
Number of families	15 524	15 702
Number of families with a family name	4905	14 849

The other major improvement was to provide a meaningful name to the families. This was done through both manual curation and computational assignment. Most of the new family name assignments utilize the PANTHER automatic subfamily name assignments, which in turn rely on protein names from UniProt (15) as described previously (19). We increased the number of families with a meaningful name from 4905 (32%) two years ago to 14 849 (95%) now (Table 2).

## ONTOLOGY UPDATES

### PANTHER protein class refactoring

The PANTHER Protein Class (PC) ontology has been completely refactored since our last update article in 2019. PANTHER PC (originally called PANTHER/X) was designed as a simple, high-level classification of the functions of proteins and protein families (1). On the PANTHER website, PANTHER PC can be used for browsing the protein families, or to visualize and compare the protein-coding gene content of different genomes.

Starting with PANTHER version 15 (released in February 2020), the PANTHER PC classification has been refactored to adhere to several principles:

1. The PANTHER PC ontology is a strict hierarchy, i.e. a functional class cannot have multiple parent classes.
2. Protein families, and not individual subfamilies or proteins, are each assigned to a PC class. PANTHER PC is therefore a classification at the level of protein families.
3. Each protein family is associated with only one PC class. The only exception to this rule is for proteins that contain multiple functional domains that belong to different classes. In other words, multiple classifications are allowed only in the case where there are two distinct, modular functions encoded by the same protein-coding gene.
4. The functional class of a protein family usually applies to all members of the family, but in some cases there may be a few family members that do not possess that function themselves. In general, the functional class reflects the ancestral, and highly conserved, function of a protein family, but there may be a few functionally divergent members.
5. Classes are designed to be as biologically informative as possible, while remaining accurate when applied to whole protein families. Relatively uninformative terms in previous versions of PANTHER PC like *nucleic acid binding* have been obsoleted. New terms have been added or modified to make important distinctions between biological functions (see below).

These properties make the PC classifications easier to navigate and interpret than the other classifications in PANTHER such as Gene Ontology. Navigation is strictly up and down the hierarchy and not across categories, and genes will not be double-counted because of their association with multiple distinct functional classes. The trade-off for this simplicity is that the functional classes in PC are relatively broad and general, and will not provide a detailed description of protein function. In addition, a few subclasses (enumerated below) could arguably appear under more than one top level class, but we have retained only the more informative one. In adopting these simplifications, the PANTHER Protein Class is complementary to the detailed Gene Ontology and Pathway classifications in PANTHER, and users can apply these different classifications in various combinations to explore sets of protein-coding genes.

In version 16, there are 210 classes in PANTHER PC. The hierarchy is no more than four levels deep. The largest number of classes at any level is at the top level, where there are 24 classes. Compared with previous versions, we have introduced 9 new classes, renamed and redefined 28 classes, and moved some of the classes within the hierarchy. The most prominent changes are:

1. Distinguishing classes of enzymes by the substrate they act on. This arrangement is more biologically informative than our previous classification by enzyme mechanism. Mechanism-based subclasses are still used underneath, but not above, the top-level classes that distinguish the following substrate types:
  - *Metabolite interconversion enzyme* (PC00262) for enzymes that act to convert small molecules into different small molecules
  - *Protein modifying enzyme* (PC00260) for enzymes that covalently modify proteins. This class excludes enzymes that function in the context of protein folding (protein-disulfide isomerases are classified under *chaperone*) and chromatin modification (histone modifying enzymes), for which there is a more biologically informative class in PC.
  - *DNA metabolism protein* (PC00009, formerly *DNA binding*) for proteins that are involved in the synthesis, modification or breakdown of DNA. Other proteins that bind DNA are now classified elsewhere, including DNA binding transcription factors, chromatin-related proteins and RNA polymerase subunits.
  - *RNA metabolism protein* (PC00031, formerly *RNA binding*) for proteins involved specifically in the metabolism of RNA. Some RNA binding proteins formerly in the more general RNA binding class have been moved to a more appropriate parent class, e.g. DNA primase is now under *DNA metabolic enzyme*.
2. Distinguishing between general and specific transcription factors:
  - New class for *general transcription factor* (PC00259), under *RNA metabolism protein*.
  - New class for *gene-specific transcription regulator* (including both DNA binding transcription factors and protein-binding co-factors).
  - Many additional new classes for subtypes of specific transcription factors (PC00244 through PC00256) to more closely match the classes in TFClass (20).
3. New top-level class for *chromatin, chromatin-binding or -regulatory protein* (PC00077, formerly *chromatin binding protein*), which now includes subclasses for histones and chromatin modifying proteins. Note that *histone modifying enzyme* (PC00261) is a new subclass here rather than under *protein modifying enzyme*, as their primary function is to modify chromatin structure, not protein structure.
4. Clear definition of *receptor* (PC00197) as *transmembrane signal receptor*. In previous versions this class was a mix of distinct functions for which the term *receptor* is also applied in biology, such as vesicle cargo receptors (now moved under *vesicle traffic protein*) and ligand-gated ion channels (now only under *transporter*).
5. Distinguishing between protein kinases according to whether they act intracellularly (under *protein modifying enzyme*), or as transmembrane receptors (under *transmembrane signal receptor*). In previous versions, protein kinases were classified by catalytic mechanism (*transferase* > *kinase* > *protein kinase*), with transmembrane receptor kinase classes having a second parentage under *receptor* (PC00197).
6. New top level class for proteins involved in translation (*translational protein*, PC00263) which groups together ribosomal proteins, translation factors and aminoacyl-tRNA synthetases.
7. Closer alignment with the Transporter Classification Database (TCDB) (21) classes for transporters. PANTHER PC now distinguishes between primary active transporters (P00068) and secondary carrier transporters (PC00258) and classifies proteins like nuclear pore subunits and ATP synthase subunits under the *transporter* branch.

**Figure 4.**
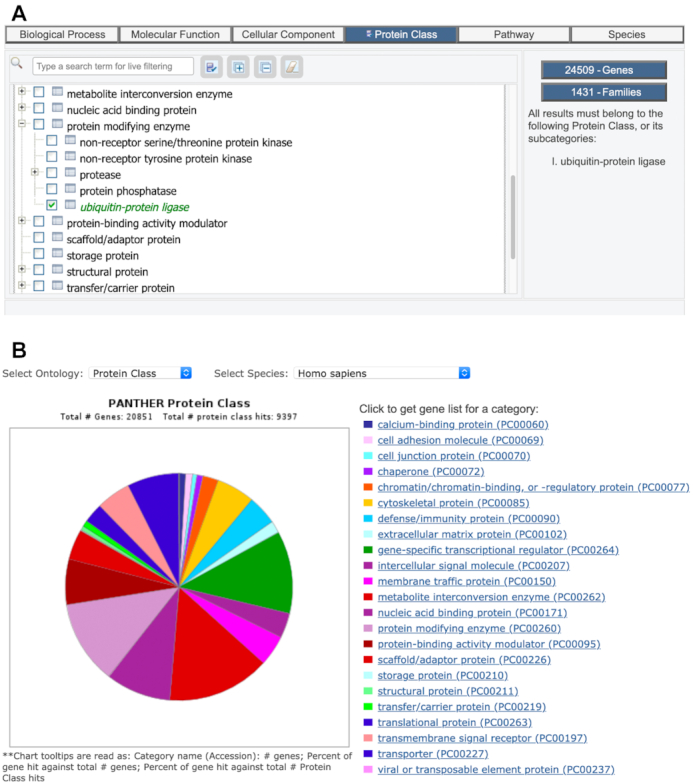
Using the Protein Class ontology. (**A**) Browsing the PANTHER data using Protein Class. Note that number of families also includes subfamilies. (**B**) Overview of entire set of protein-coding genes in a genome.

The main use cases for the PC ontology are in browsing the collection of gene families in PANTHER, and in getting a clear overview of an entire protein-coding genome (or comparing more than one genome). Figure 4 illustrates these use cases, with screen shots of the actual tools available on the PANTHER website. Users can also use PC classifications to visualize or subset any gene list, including those created from GO or pathway classifications, or a user-specified gene list that was uploaded to the PANTHER website.

In the past two years, we have completely re-curated the associations between PANTHER families and PANTHER PC classes, including reviewing all classifications from previous versions. In PANTHER version 16.0, 6101 of the 15 702 families (39%) are associated with a Protein Class. These tend to be the larger families, resulting in a large fraction of protein-coding genes with a PC assignment. In PANTHER version 16, 58% of human genes (12043) are in families with a PC assignment. Users should note that a family can be associated with a PANTHER PC class at any level, and while we strive to make the classification as specific as possible, many protein families may appear only in a more general class. If users see a classification they think might be incorrect or too general, we encourage them to notify us so we can prioritize it for review.

### PANTHER GO-slim update

The new PANTHER GO-slim was updated from the latest phylogenetic annotations provided by the GO Phylogenetic Annotation project (12). It contains 3336 terms, with 2234 biological process, 557 molecular function and 542 cellular component terms. The number of classes has increased by 289 terms compared to the one reported in our last update in 2019 (13), due to the number of additional GO terms that have been used by the GO Phylogenetic Annotation project. The ontology can be downloaded from http://data.pantherdb.org/PANTHER16.0/ontology/panther_slim.obo.

## NEW SERVICES AND TOOLS

### New PANTHER services

The era of big data has been driven by increases in the speed and resolution of data acquisition methods, and PANTHER is no exception. Automated methods to analyze data has become a necessity for researchers and biologists for comprehensive studies of biological systems. In addition to data analysis, scientists are no longer interested in only raw data, but also data that has been categorized and curated. PANTHER datasets have also grown, and there is an ever-increasing number of users who are interested in accessing it to further their research. PANTHER services have been created to address the exponential growth in data by providing tools to retrieve and analyze data. It gives users programmatic access to PANTHER's expert-curated data and analytical tools via RESTful Web Services (22). Supporting programmatic access allows researchers to easily incorporate PANTHER into existing or new workflows for scientific discoveries. The services are described using the OpenAPI Specification (link http://api.pantherdb.org) which is a broadly adopted industry standard.

As indicated by system usage statistics, PANTHER is primarily used in three ways: (i) to compare lists of genes to determine if any biological processes are under or over represented in a list, (ii) to retrieve annotation information for a given set of genes and (iii) to retrieve information about related genes based on evolutionary history.

Careful consideration was given to creating service endpoints that could be incorporated into meaningful workflows. In order to support the various use cases, three main categories of services have been created: Utilities, Data Mapping and Phylogenetic Tree services (Table 3). The Utilities module contains the widely used overrepresentation service, which compare a list of genes to a reference list to statistically determine over- or under-representation of a specified ontology class. By default, the system uses Fisher's exact test with FDR correction, but there is also an option to use the Binomial test. Both methods have options for FDR correction, Bonferroni correction or no correction. This service supports various annotation data sets: Complete GO (three aspects, listed above), PANTHER GO-slim (three aspects), PANTHER Protein Class, PANTHER Pathway, and Reactome Pathways.

**Table 3.** Summary of PANTHER Services

Services	Tools	Description
Utility	Statistical overrepresentation test	Fisher's Exact test with option of FDR or Bonferroni correction
	Ortholog/paralog service	Returns orthologs from a user specified organisms or paralogs for an input gene list
	Homolog position service	Given a gene and an amino acid position, returns a list of homologs for the gene from a user specified organisms and corresponding amino acid positions
Data Mapping	Gene list classification	Returns functional classification of an input gene list
Phylogenetic Tree	Tree service	Returns the tree topology and its attributes of an input of PANTHER family ID
	MSA service	Returns the multiple sequence alignment of an input PANTHER family ID. The alignment includes both leaf sequences and internal nodes
	Family ortholog	Returns all orthologs for sequences for an input PANTHER family ID
	TreeGrafting	Returns a tree topology of a family tree with grafted protein sequence

Annotations associated with ‘complete’ Gene Ontology classes are updated monthly from the official Gene Ontology release. This group of PANTHER web services also includes modules to search for orthologs and other homologs of genes.

The Data Mapping service allows users to upload a list of genes and get back information about expert curated annotations and pathways that have been assigned to the input list of genes.

PANTHER trees contain groups of genes based on evolutionary history, and given that PANTHER currently supports over 142 genomes, it was imperative that users were equipped with tools to focus on specific taxa based on their field of research. The PANTHER species tree (http://pantherdb.org/panther/speciesTree.jsp) allows users to select higher order taxonomic groups or lower level species of interest and obtain associated lists of families and taxonomies. The list of families can be used as input with the Phylogenetic Tree services module to only retrieve trees containing species of interest. Furthermore, the NCBI taxonomy identifiers can be specified with the Phylogenetic Tree services to include only proteins from selected taxa. A service to determine the list of orthologous genes in a family is also available as part of this module.

A grafting module (see TreeGrafter description below) is also available that allows users to specify a protein sequence and retrieve the best matching homologous family and graft in the best location in the tree. Additionally, it also returns the associated annotations associated with the specified sequence.

Due to the nature of constantly changing data, annotations and tools, all services include versioning and date information for ensuring that analysis results can be replicated by researchers, peers and reviewers.

### TreeGrafter software for classifying user-specified protein sequences

TreeGrafter is a software package for classifying a protein sequence by ‘grafting’ it onto a reference protein family tree (11). The algorithm proceeds in three steps: (i) scoring a sequence against a library of family HMMs, (ii) adding the new sequence to the reference multiple sequence alignment for the highest-scoring family and (iii) determining the most parsimonious graft point for the new sequence, relative to other sequences in the reference tree. TreeGrafter has been shown to be more accurate than the subfamily HMM scoring that users of PANTHER have been applying for over 20 years.

**Figure 5.**
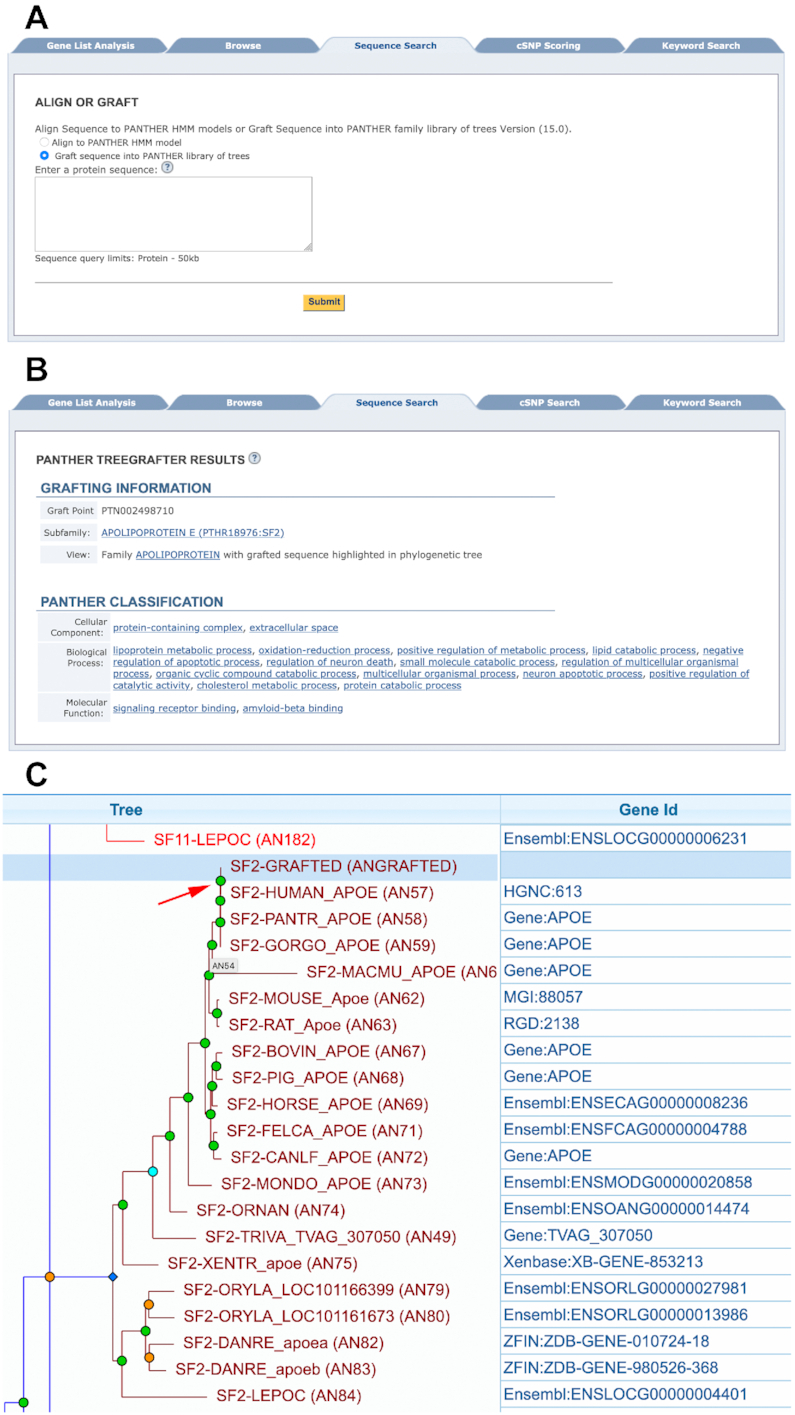
Using TreeGrafter to classify a new protein sequence. (**A**) Users can classify new sequences using HMMs, or TreeGrafter. (**B**) TreeGrafter results show subfamily assignment, and GO terms that depend on the graft point in the tree. (**C**) Users can view the grafted sequence in the context of the reference phylogenetic tree. The new sequence is an additional leaf in the tree (marked ‘ANGRAFTED’ and highlighted in blue), with the new internal node (pointed by a red arrow) induced by the grafting (marked ‘ANINDUCED’).

Starting with PANTHER version 15 (released in early 2020), users can now apply TreeGrafter for classification of new sequences. This is an additional option for users; the older subfamily HMM searching is still available. TreeGrafter can be accessed interactively on the PANTHER website, from the ‘Sequence Search’ tab on the homepage (Figure 5A). Annotations for a new sequence depend on the graft point on the reference tree, and include PANTHER subfamily, and manually-annotated GO terms on tree branches made by the Gene Ontology Phylogenetic Annotation project (12) (Figure 5B). In addition, the phylogenetic tree showing the graft point of the sequence can be visualized using the PANTHER TreeViewer tool (Figure 5C). TreeGrafter for classifying a single sequence can also be accessed via web services (see above), or downloaded as a command-line tool for use at large-scale (https://github.com/pantherdb/TreeGrafter). Note that TreeGrafter annotations are updated with every PANTHER release.

## ENHANCER-GENE LINKS

### Enabling annotation of genetic variants in non-coding enhancer regions

**Figure 6.**
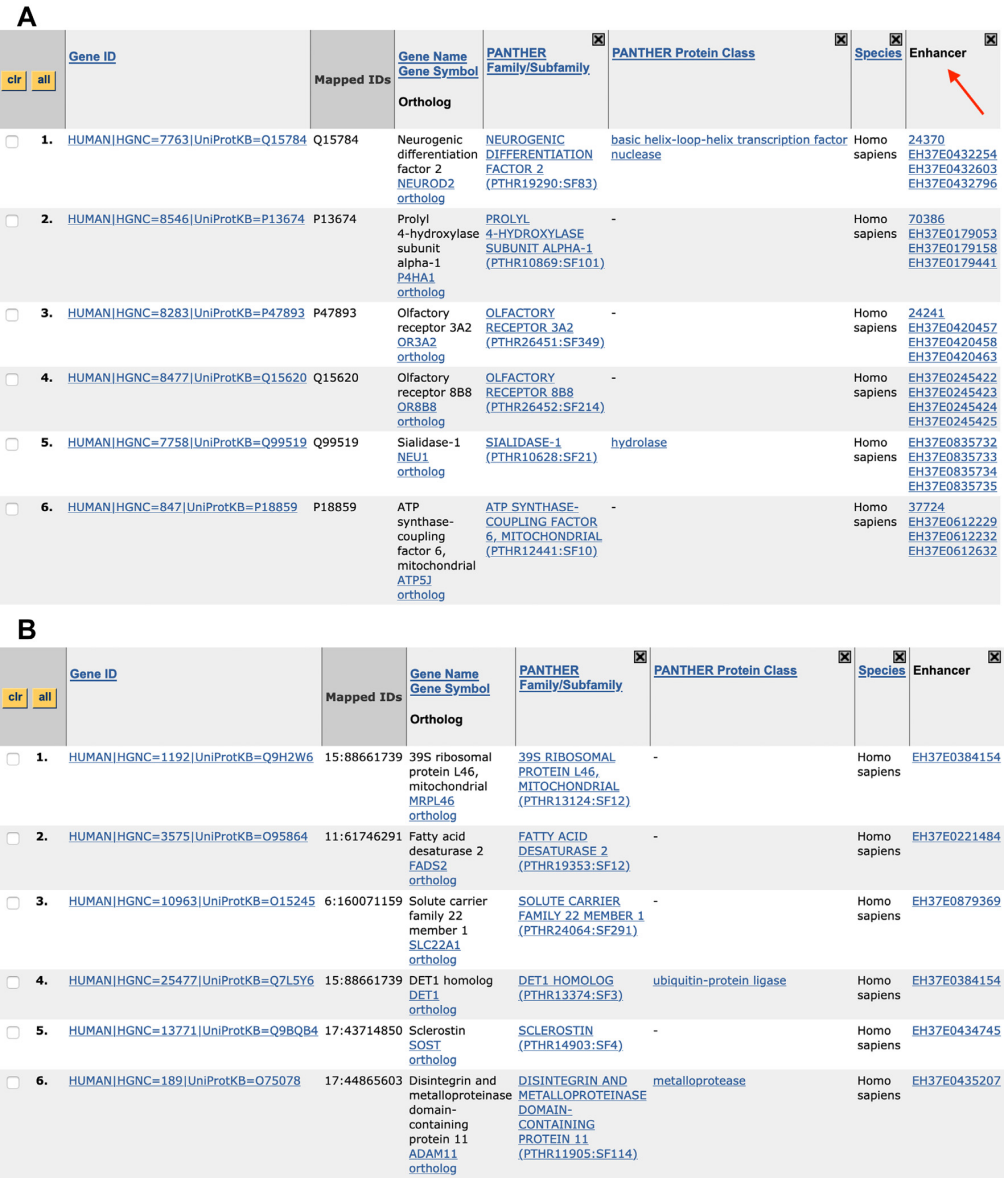
PANTHER now includes PEREGRINE enhancer-gene links data. (**A**) A screenshot of a gene list page after the user queries the PANTHER system with a list of genes (UniProt IDs in Mapped IDs column). The enhancers associated to each gene are listed in the Enhancer column (red arrow), which is not shown by default but can be added by clicking on a link above the list header (not shown). (**B**) A screenshot of the PANTHER gene list page when a user submits a VCF file. The coordinates of the variants are listed in the Mapped IDs column. PEREGRINE will map the variants to the enhancers (Enhancer column) as well as the gene(s) that is regulated by the enhancers (Gene ID column).

In our 2017 update (23), we described the extension of the gene list analysis tool to allow direct upload of human genetic variants. The tool initially considered only variants in the protein-coding regions of the human genome. However, the majority of genetic variants are in the non-coding regions of the genome. Enhancers are short DNA elements in those regions that function primarily as clusters of transcription factor binding sites that are spatially coordinated to regulate expression of one or more specific target genes. PEREGRINE (**P**redicted by **E**xperimental **R**esults: **E**nhancer-**G**ene **R**elationships **I**llustrated by a **N**exus of **E**vidence) is an enhancer to gene link database (www.peregrineproj.org) with genome-wide characterization of regulatory connections between enhancers and target genes (submitted). It incorporates publicly available experimental data from ChIA-PET, eQTL and Hi-C assays across 78 cell and tissue types to link 449 627 enhancers to 17 643 protein-coding genes. The PEREGRINE human enhancer-gene links have now been added to the PANTHER database. There are two ways for users to access the data. The first is to find the enhancers that are associated to a particular gene by querying the target gene using the existing gene search function in PANTHER. An enhancer column can be added to the display (by clicking on the ‘view enhancer data’ link) to retrieve the list of enhancers (Figure 6A). The second is to submit a list of genetic variants (in VCF format) and retrieve a list of enhancers these variants are located in, and the target gene(s) each enhancer may interact with (Figure 6B). The details about the enhancer-gene links, including tissue information and assays that support the links, can be viewed by clicking each identifier in the Enhancer column.

## CONCLUSIONS

PANTHER development has been focused in two main directions, and we expect these trends to continue in the future. The first direction is the continual increase in the coverage of protein-coding gene diversity across the tree of life. In the past decade, the set of PANTHER reference trees has expanded from ∼6500 families covering 48 fully-sequenced genomes in PANTHER version 7 (24), to ∼15 500 families covering 142 genomes in the current version. At the same time, we have continued to increase the quality of families, refining family boundaries and improving the phylogenetic trees based on ongoing review and semi-automated curation. PANTHER has become a suitable tool for analysis of lists of genes, and full genomes, from organisms across the tree of life.

The second direction of development is the increase in coverage and specificity of the annotations. PANTHER has expanded from family, subfamily and Protein Class annotations, to including GO terms and pathways, and now human enhancer regions. Specificity of annotation has dramatically improved. PANTHER was initially based on identifying subtrees containing genes of clearly diverged functions, and constructing HMMs that distinguished different subfamilies (1). However, this approach becomes difficult to scale as annotations become more specific, and are confined to ever-smaller subtrees. Since 2010, individual nodes in PANTHER trees have been annotated with gain and loss of function annotations (12), enabling functional conservation and divergence to be represented at whatever level of detail is required, on a case-by-case basis. Functions are annotated using GO terms, as well as signaling and metabolic pathways. Using TreeGrafter, these fine-grained annotations can be transferred to sequences that are not in the reference tree by grafting directly onto a specific branch in the tree, rather than using subfamily HMMs to identify an approximate clade within the tree. Currently, TreeGrafter uses the tree graft point to infer the PANTHER subfamily, and PANTHER GO-slim annotations. In the future, we expect that tree grafting will also enable additional applications of the reference tree, including identifying the taxonomic group to which a sequence may belong, and inferring its orthologs in other organisms.
